# First person – Sek-Shir Cheong

**DOI:** 10.1242/dmm.050556

**Published:** 2023-11-03

## Abstract

First Person is a series of interviews with the first authors of a selection of papers published in Disease Models & Mechanisms, helping researchers promote themselves alongside their papers. Sek-Shir Cheong is first author on ‘ A method for TAT-Cre recombinase-mediated floxed allele modification in *ex vivo* tissue slices’, published in DMM. Sek-Shir is a research associate in the lab of Dr Charlotte Dean at Imperial College London, London, UK, investigating mechanisms of lung repair and regeneration using 3D living lung tissues.


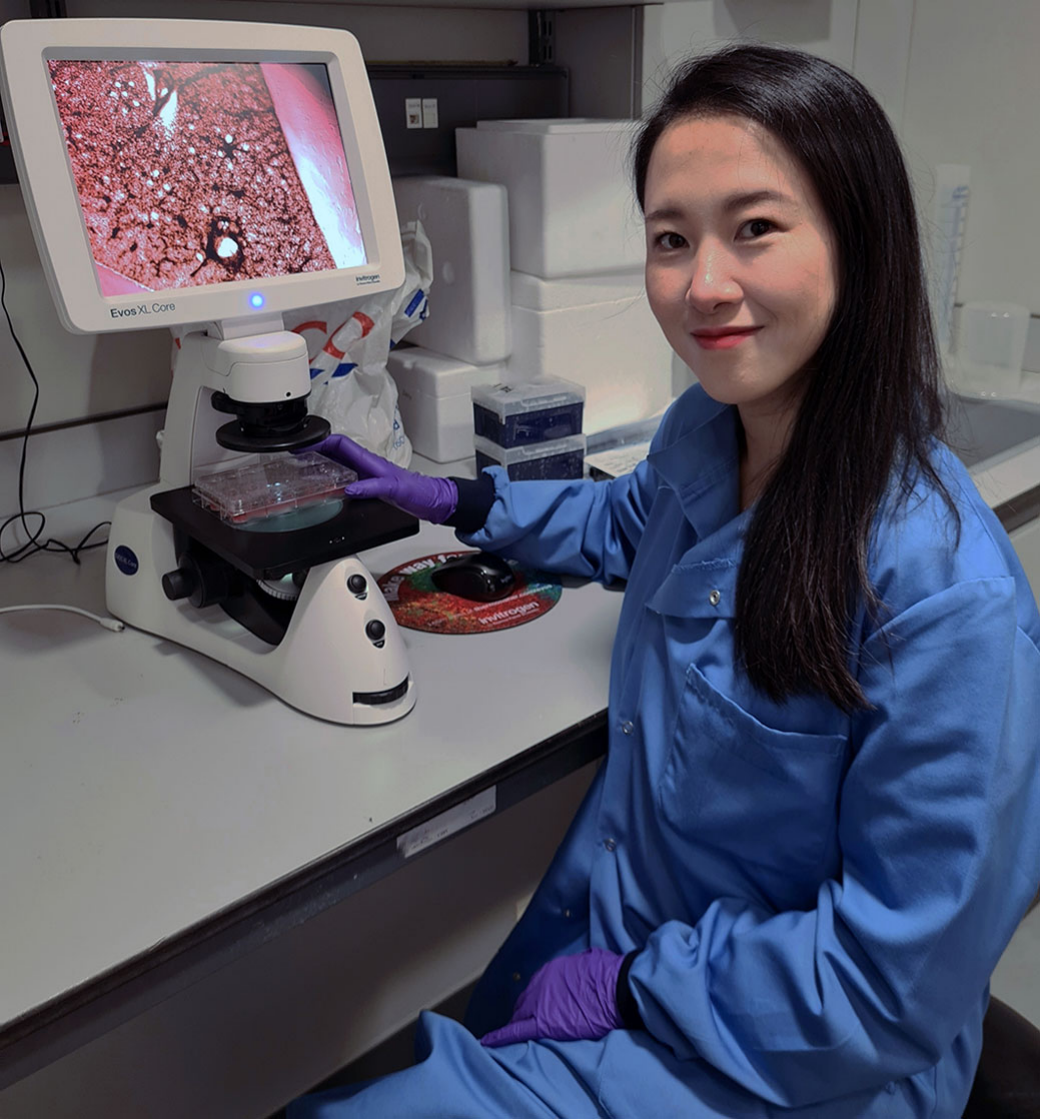


**Sek-Shir Cheong**

**How would you explain the main findings of your paper to non-scientific family and friends?**

In the world of medical research, understanding how genes work is like solving a puzzle that holds the secrets to diseases and their potential treatments. Trying to understand what role a particular gene has in our internal organs is difficult; it is usually not possible at all in humans and instead we use specially adapted (genetically modified) animals, in most cases mice, that allow us to discover what individual genes do. However, this presents challenges, both in terms of time and resources. We addressed this challenge by developing a groundbreaking method, called TAT-Cre recombinase-mediated floxed allele modification in tissue slices (TReATS), which allows us to modify genes in living pieces of lung tissue taken out of the body. These tissue slices are effectively ‘mini-lungs’. What's thrilling about this method is that it helps us investigate how genes work in the lung much more easily and comprehensively than previous methods, whilst vastly reducing the number of laboratory animals involved and the time that experiments take.

**What are the potential implications of these results for your field of research?**

The potential implications of our novel method are profound for our field of research. Lung diseases claim the lives of approximately 7.6 million people annually, making them a leading global cause of mortality. Despite the staggering toll, there remains no definitive cure for most common lung diseases. By simplifying the process of modifying genes in lung tissue, so that this can be done outside the body, far fewer mice are needed and we also save a lot of time, which makes our research much more efficient. One other important outcome of our novel method is that it enables us to modify genes that, when mutated, typically lead to early mortality, preventing us from studying their role in the lungs in a living animal. This means we can study many lung diseases more effectively and hopefully discover new ways to treat them. In short, our methodology opens up new possibilities for research in the field of lung biology and disease.

**What are the main advantages and drawbacks of the experimental system you have used as it relates to the disease you are investigating?**

Our experimental system offers a multitude of advantages. Not only does it save time and resources in dissecting the role played by specific genes in lung diseases, but it also allows us to study genes that cause premature death when they go wrong. However, a drawback is that our method relies on keeping the lung tissue alive outside the body, and, at the moment, there are limitations in terms of how long we can keep the tissue slices healthy to study them. However, working with collaborators, the Dean Lab is actively exploring ways to significantly extend the lifespan of lung slices in culture and the preliminary results are very encouraging.

The extent to which the TReATS method successfully modifies genes throughout all the cell populations and regions of the lungs simultaneously far surpassed our initial expectations.

**Figure:**
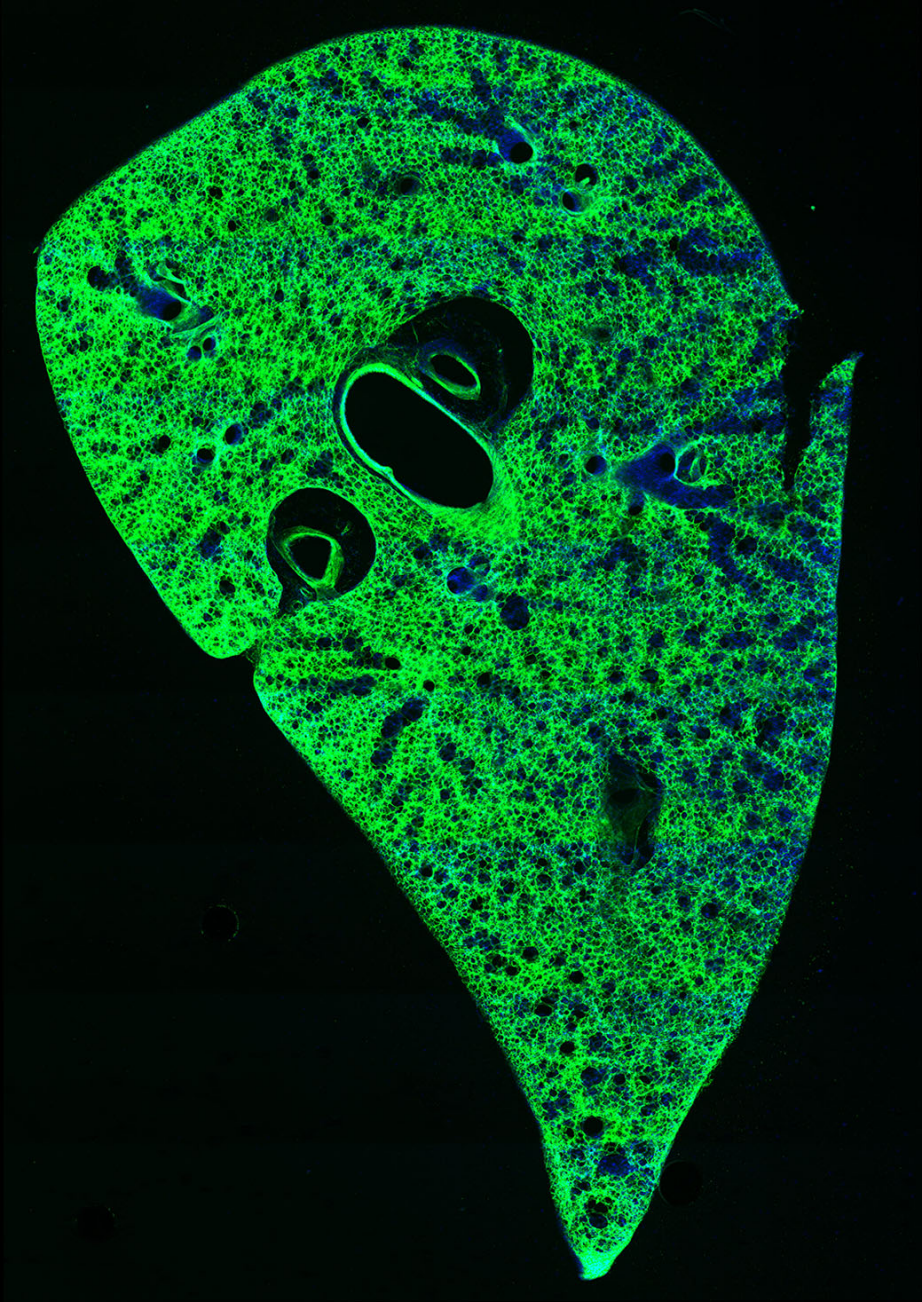
A glowing mouse precision-cut lung slice with activated *EYFP* gene using the Dean Lab's innovative TReATS method.

**What has surprised you the most while conducting your research?**

What truly amazed us during our research was the sheer effectiveness, robustness and simplicity of our novel method. The extent to which the TReATS method successfully modifies genes throughout all the cell populations and regions of the lungs simultaneously far surpassed our initial expectations.

**What do you think is the most significant challenge impacting your research at this time and how will this be addressed over the next 10 years?**

Right now, the most significant challenge impacting our research is studying the long-term effects of gene changes in lung tissue. When we modify genes to understand their role in lung diseases, we can see the immediate or short-term effects. However, understanding how gene changes affect the lung over an extended period can be challenging when working with tissue outside the body. Over the next decade, we hope to address this by further improving our methods to keep the tissue viable for even longer. This means we can unveil the long-term impacts of gene changes, gaining a deeper understanding of lung disease and efficacy of potential treatments. Also, we are committed to expand this method to other organs.

**What changes do you think could improve the professional lives of scientists?**

In my opinion, Imperial College London is excellent at providing not just personal and professional support, but also offering an academically inspiring environment for scientists. However, better funding and resources for research, as well as more opportunities to build a sense of community among scientists could make a difference in our professional lives.

**What's next for you?**

What's next for us is to continue refining and expanding the use of the TReATS method. We aim to use it to study various aspects of lung biology and diseases. Additionally, we hope to collaborate with other researchers to apply this method to different organs and fields of study, potentially leading to exciting new discoveries in science and medicine.
